# The Tobacco Control Scale as a research tool to measure country-level tobacco control policy implementation

**DOI:** 10.18332/tid/128318

**Published:** 2020-11-03

**Authors:** Ariadna Feliu, Esteve Fernández, Antoni Baena, Luk Joossens, Armando Peruga, Marcela Fu, Cristina Martínez

**Affiliations:** 1 Tobacco Control Unit, WHO Collaborating Centre for Tobacco Control, Institut Català d’Oncologia, Barcelona, Spain; 2 Tobacco Control Research Group, Institut d’Investigació Biomèdica de Bellvitge, Barcelona, Spain; 3 Faculty of Medicine and Health Sciences, Universitat de Barcelona, Barcelona, Spain; 4 Consortium for Biomedical Research in Respiratory Diseases (CIBERES), Madrid, Spain; 5 eHealth Center, Faculty of Health Sciences, Universitat Oberta de Catalunya, Barcelona, Spain; 6 Tobacco Control Expert, Leuven, Belgium; 7 Center for Epidemiology and Health Policies, Clínica Alemana, School of Medicine, Universidad del Desarrollo, Santiago de Chile, Chile; 8 Philip R. Lee Institute for Health Policy Studies, University of California San Francisco, San Francisco, United States

**Keywords:** tobacco, tobacco control policies, epidemiology, research

## Abstract

**INTRODUCTION:**

The Tobacco Control Scale (TCS) was designed for advocacy purposes but has also been used as a research tool. In the present study, we characterized TCS use, its limitations and strengths, and critically assessed its use as a research instrument.

**METHODS:**

We conducted an extensive search of the biomedical databases PubMed and Web of Science for the keyword ‘tobacco control scale’ in all fields. The search was limited to studies published in the period March 2006 to December 2019. Out of 69 hits, 32 studies met the inclusion criteria. Two reviewers independently extracted information from each publication regarding their general characteristics, publication and research aspects, and the characteristics of the use of the TCS.

**RESULTS:**

We found that researchers have used the TCS as a tool to monitor tobacco control policies mainly in cross-sectional observational studies with ecological and multilevel designs directed to advocacy and the promotion of further research. Different outcomes, such as smoking prevalence and quit ratios, have been associated with tobacco control policy scores. The main reported limitations of the TCS were a low variance across countries and a failure to express enforcement and to incorporate the most recent legislation.

**CONCLUSIONS:**

The TCS has been commonly used to assess differences in outcomes according to tobacco control policies. However, there are still areas for improvement in its use in research regarding the lack of comparability of TCS scores across time. The lessons that have been learned should be used to adapt and expand the TCS overseas.

## INTRODUCTION

Effective tobacco control policies help denormalize smoking, decrease smoking prevalence^1^, and reduce morbimortality attributable to tobacco^2^. Many efforts have been made globally to tackle the tobacco epidemic^3^, stimulated by the enforcement of the Framework Convention on Tobacco Control. In the European Union (EU), the Tobacco Products Directive has driven the application of stringent tobacco control policies to reduce tobacco use and its negative consequences on health. However, the implementation and enforcement of tobacco control policies still vary greatly across Europe^4^.

Among the initiatives to monitor the implementation of tobacco control policies in Europe, Joossens and Raw^5^ developed the Tobacco Control Scale (TCS) in 2006. The TCS score is determined by a questionnaire based on six cost-effective policy interventions that should be prioritized according to the World Bank. These measures include taxation, smoke-free policies, public spending in information campaigns, advertising bans, health warnings, and treatment. The score assigned to each of these components is weighted by its reported evidence-based effectiveness. Therefore, the score attributed to each country increases with the strength of tobacco control policies up to a maximum of 100 points, indicating full implementation^5^.

At its inception, the aim of the TCS was to monitor the progress in tobacco control in Europe at a national level by comparing the performance of countries by their ranking^5^ and to inform the agenda by highlighting the policy components for which progress is lacking, as well as the countries or regions most affected by such delays^6^. Since 2006, the TCS has been updated every three years (available at www.tobaccocontrolscale.org).

Evaluating the impact of tobacco control policies among the population has become an important research area; thus, the TCS has been used as a research tool to measure the implementation of tobacco control policies, though it was not designed for such purposes. However, little is known about the use of the TCS by the tobacco control research community and its advantages and disadvantages as a research tool. Therefore, our aim was to characterize the use of the TCS by researchers and its main limitations and strengths as a research tool in order to critically assess its use as a research instrument.

## METHODS

### Data sources

We performed an extensive literature search in the online databases PubMed and Web of Science to identify publications that have used the TCS score(s) as an independent or dependent variable from 27 March 2006, when the first TCS was published, until 1 December 2019. The search was conducted using ‘tobacco control scale’ as the keyword in all fields without any other restrictions to ensure a very sensitive search. The Ethics and Clinical Research Committee of the Hospital Universitari de Bellvitge approved this study (PR247/18).

### Study selection

**Figure 1:**
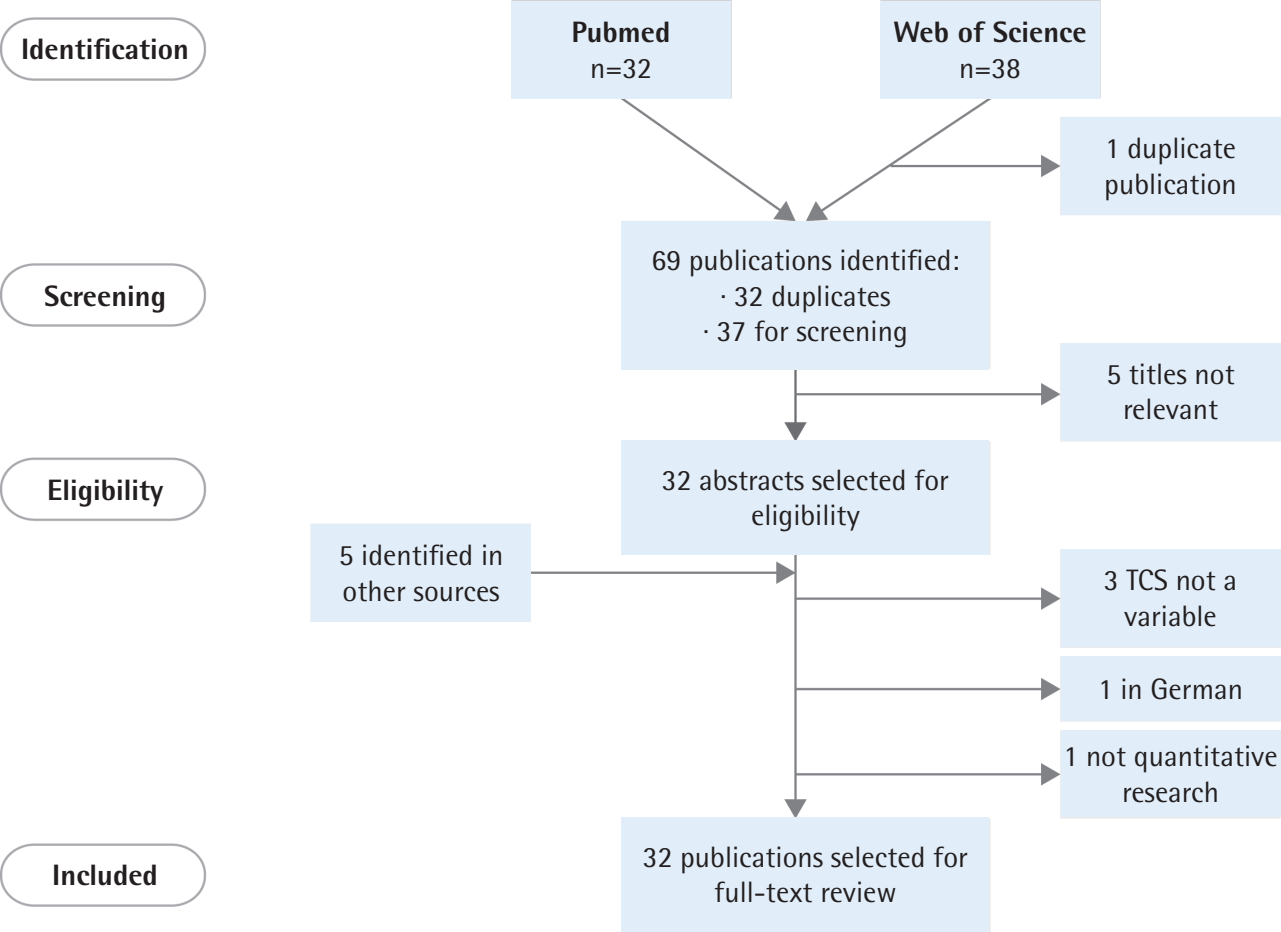
Flowchart of the selection process of publications for full-text review

We identified 69 publications (32 duplicated in both databases). After removing duplicates, two researchers (AF and AB) screened the titles and abstracts, obtaining 32 studies. The inclusion criteria were quantitative research and inclusion of the TCS score(s) (as dependent or independent variable) in the analysis. We found 27 eligible publications (Figure 1). We completed our search by manually reviewing the reference lists of the selected papers and by conducting the same search in Google Scholar (www.scholar.google.com; with search terms in English). These additional searches provided five new publications that met the inclusion criteria and the full-texts were reviewed.

Therefore, we finally included 32 publications that used the TCS score(s) as a dependent or independent variable.

### Data extraction

A detailed protocol and Microsoft Access^®^ database were designed to extract and register the information from each publication. The evaluation protocol was developed by three researchers who are experts in tobacco control (AF, CM, and EF). The protocol describing the main objectives, information sources, search strategy and eligibility criteria, and the data collection was reviewed and approved by all researchers. All variables for which data were described in the publications’ Methods sections were listed.

Two reviewers independently extracted the data according to the protocol (AF and AB). If any discrepancies emerged, the reviewers discussed the papers until agreement was reached and, when no consensus was met, divergences were solved by discussing them with a third reviewer (CM). The evaluation process was conducted in January 2020.

The extracted information included general characteristics, publication characteristics, research characteristics, and characteristics of the use of the TCS (Table 1).

**Table 1:** Summary of the information extracted (variables and categories) from each publication

	*Variables*	*Categories*
**General**	Author surname and initials, institutional affiliation, and country of affiliation of the first author	
	Funding	Yes, no, or not declared; and if yes, private, public, or both
	Conflicts of interest	Yes, no, or not declared
**Publication**	Type of publication	Research paper, brief paper, review, letter to the editor, editorial, comment, or other
	Publication year	
	Journal	
	Open Access	Yes or no
**Research**	Objective of the study	
	Type of design	Observational or experimental; cross-sectional or longitudinal
	Type of study data	Individual, ecological, or multilevel, with individual data from surveys as first level unit with TCS score by country as second level of aggregated data
	Main results	( literals )
	Limitations (specifically, those related to the use of the TCS as a tool to monitor tobacco control policy implementation)	( literals )
	Main conclusions and their purpose	For advocacy (when directly addressed to stakeholders and policymakers), to undertake further research on the topic, or both
**Use of the Tobacco Control Scale (TCS)**	Type of variable	Dependent, independent, or both
	Year of the TCS report	
	Source of the TCS score(s)	Primary source, when publications included the TCS score(s) from the original reports; or secondary source, when publications included TCS data from other publications in which case the alternative data source was recorded
	Total score	Yes or no
	Individual components score(s)	Yes or no; and if yes, which components were included
	Countries from the original TCS report(s)	Yes or no; and if not, we recorded the number of countries included

## RESULTS

The 32 publications were published between 2008 and 2019. More than two-thirds (n=23; 71.9%) were published by research groups from the Netherlands (n=8), Spain (n=9), and the United Kingdom (n=6). Almost all (n=30; 93.8%) were original articles published in peer-reviewed journals, 12 of which were Open Access (37.5%). In addition, almost all declared no conflicts of interest (n=29; 90.6%) and were financially supported (n=26; 81.3%) with public funds (n=19; 73.1%), both public and private funds (n=5; 19.2%), or private funds (n=2; 7.7%).

Most of the publications (n=31) were observational studies; 16 used ecological data (50%) with the country as the unit of analysis and 16 used multilevel data (50%) with individual data from surveys as the first-level unit and TCS score by country as second-level aggregated information. Regarding the study design, 23 of the publications were crosssectional studies (78.6%). Most of the articles (n=24; 75%) (Table 2) included the TCS score(s) as an independent variable from primary reports, whereas 10 publications (31.3%) (Table 3) used the scores from secondary sources that calculated a new score based on the TCS methodology. Overall, 87.5% (n=28) of the publications used the overall TCS score by country and 65.6% (n=21) used the individual policy component scores.

**Table 2:** Main characteristics of the studies that used the original TCS reports as primary data sources (N=22)

*Reference, location*	*Objective*	*Study design*	*Use of TCS (Type of variable, original data, total and components score)*	*Main results*	*Limitations*	*Conclusions*
Feliu et al.^8^ 2019, Spain	To empirically evaluate whether the hardening hypothesis can be confirmed at the population level in the 28 EU member states, and to analyze the determinants of hardcore and light smoking considering both individual and contextual country-level characteristics	Observational, multilevel, time-trends	Independent variable, original data, only total score	Hardening smoking is not increasing in EU member states where smoking prevalence is decreasing. Odds of being a hard-core smoker are higher among middle-aged men of lower class and lower in countries with higher TCS scores	No limitations reported about the TCS as a variable	Advocacy
González-Marrón et al.^13^ 2019, Spain	To explore the association between the implementation of tobacco control policies and the risk of lung cancer in the EU	Observational, multilevel, cross-sectional	Independent variable, original data, only total score	Significant inverse correlation between TCS 2010 and the proportion of former and ever smokers at high risk of lung cancer according to NELSON criteria	The cross-sectional design of TCS limits the validity of the study to establish causation	Advocacy and research
Feliu et al.^4^ 2019, Spain	To assess the midterm association of tobacco control policies on smoking prevalence and quit ratios among 27 EU countries	Observational ecological, and cross-sectional	Independent variable, original data, total score and by components	In EU27, countries with higher scores in the TCS has lower prevalence of smokers, higher quit ratios, and higher relative decreases in their prevalence of smokers over the last decade	The TCS does not score the level of enforcement except for smoke-free policies and the score may not fully reflect tobacco control policies implemented in subsequent years. The ranking of countries according to TCS scores has been relatively consistent across different editions	Advocacy and research
Diez-Izquierdo et al.^7^ 2018, Spain	To evaluate the correlation between tobacco control policies and the prevalence of preterm births and low birthweight in the European countries	Observational,ecological, and cross-sectional	Independent variable, original data, total score and by components	The TCS score negatively correlated with the prevalence of preterm births for <37 weeks and <32 weeks and the prevalence of low birthweight (<2500 g) in European countries in 2010	No limitations reported about the TCS as a variable	Advocacy
Lidón-Moyano et al.^18^ 2018, Spain	To describe the acceptability of some tobacco product regulations and to explore their relationship with tobacco control legislation levels in Europe	Observational,ecological, and cross-sectional	Dependent and independent variable, original data, total score and by components	Strong support for tobacco product regulations was observed. A positive relationship was found between TCS scores and support for tobacco product regulations at both the ecological and individual level	No limitations reported about the TCS as a variable	Research
Filippidis et al.^22^ 2017, UK	To examine associations between median cigarette prices, price differentials between cigarette brands, and infant mortality	Observational, ecological, and longitudinal	Independent variable, original data, only by components score	Larger differences between median and minimum cigarette prices were associated with increased rates of infant mortality. Median price increases during the study period were associated with 9208 fewer infant deaths, but a further 3195 infant deaths could have been avoided if no price difference was observed between minimumpriced and median-priced cigarettes	No limitations reported about the TCS as a variable	Advocacy
Filippidis et al.^24^ 2017, UK	To explore factors associated with self-reported exposure of the EU population to tobacco products and electronic cigarette advertising	Observational, multilevel, and cross-sectional	Independent variable, original data, only by components score	40.0% and 41.5% of respondents reported seeing any e-cigarette and tobacco product advertisement within the past year. Smokers, males, younger respondents, those with financial difficulties, people who had tried e-cigarettes, and daily internet users were more likely to report it. Respondents in countries with more comprehensive advertising bans were less likely to selfreport exposure to any tobacco, but not to e-cigarette advertisements	No limitations reported about the TCS as a variable	Research
Kuipers et al.^23^ 2017, the Netherlands	To estimate the impact of introducing sales restriction laws in European settings and to test whether the impact of the laws differed between adolescents of high and low socioeconomic position	Experimental, multilevel, and cross-sectional	Independent variable, original data, total score by components	No decrease in smoking in countries that introduced sales restrictions for minors (2007–2011) compared to countries that introduced these restrictions earlier (before 2007). Sales restrictions were associated with a stronger decrease in perceived ease of the obtainability of cigarettes. The results were similar for adolescents of high and low socio-economic position	No limitations reported about the TCS as a variable	Advocacy
Lidón-Moyano et al.^12^ 2017, Spain	To analyze the correlation between the implementation of tobacco control policies and tobacco consumption, particularly rolling tobacco and e-cigarettes, and the intent to quit smoking in 27 countries of the EU	Observational, ecological, and cross-sectional	Independent variable, original data, total score and by components	An inverse correlation between TCS score and prevalence of smoking of conventional cigarettes and a positive correlation with the intent to quit smoking within the past 12 months. The correlation between TCS and secondhand smoke (SHS) exposure at work was negative. Significant negative correlation between TCS score and the prevalence of having ever tried a waterpipe	The 2-year gap between the measure of the TCS and the Eurobarometer survey does not allow detection of the effect of measures adopted between 2010 and 2012	Advocacy
Allen et al.^35^ 2016, UK	To evaluate the potential effectiveness of maximizing the TCS score for the UK using a model stratified by socio-economic circumstances and to illustrate health improvements associated with reduced smoking prevalence	Observational, ecological, and longitudinal	Independent variable, not original data, only total score	Improvements in tobacco control policies towards maximum TCS score could substantially reduce smoking prevalence and reduce health-related inequalities	Implementation was not considered	Advocacy
Ferketich et al.^1^ 2016, USA	To determine the relationship between the TCS score and the prevalence of in-home smoking bans and beliefs on other tobacco control policies	Observational, multilevel, and cross-sectional	Independent variable, original data, total score and by components	The TCS score was correlated with the prevalence of inhome smoking bans. Four of the individual contributions to the TCS scale (price, public campaigns, smoking bans and health warnings) were significantly related to inhome smoking bans	No limitations involving the use of TCS	Advocacy and research
Pförtner et al.^10^ 2016, Germany	To address to what extent different measures of the TCS are associated with smoking in adolescence in 29 European countries and how the association between tobacco control policies and smoking varies by family affluence	Observational, multilevel, and cross-sectional	Independent variable, original data, only by components score	Tobacco control policies did not strongly interact with Family Affluence Scale (FAS) predicting adolescent smoking. For boys, prevalence of smoking decreased with higher tobacco price regardless of the socio-economic background. For girls, no difference was found in smoking prevalence by FAS	a. The limited number of observations at country level and the low variance of some tobacco policies across countries may reduce the robustness of parameter estimates; b. Analyzed data from the TCS has changed since 2006	Advocacy and research
Filippidis et al.^15^ 2016, UK	To explore whether exposure to SHS among non-smokers in the EU showed any association with sociodemographic factors and/or the extent of national tobacco control policies	Observational, multilevel, and cross-sectional	Independent variable, original data, total score and by components	29.0% of non-smoking participants reported being exposed to SHS in indoor areas. Males and individuals with difficulties paying bills had significantly greater odds of being exposed. For every unit increase of a country’s score on the Smoke-free Component of the TCS, the probability of reporting exposure to SHS increased in bars, restaurants, and workplaces	No limitations reported about the TCS as a variable	Advocacy
Martínez- Sánchez et al.^14^ 2014, Spain	To evaluate the correlation between the implementation of tobacco control policies and smoking prevalence in private venues in the 27 countries of the EU	Observational, ecological, and cross-sectional	Independent variable, original data, total score and by components	No correlation was found between the implementation of smoke-free legislation at work and in public places and an increase in prevalence of smoking in private venues in the EU. More developed smoke-free policies positively correlated with a high prevalence of smoke-free houses	No limitations reported about the TCS as a variable	Advocacy and research
Rughinis et al.^19^ 2014, Romania	To investigate the relationship between the number of cigarettes smoked daily and habits and beliefs concerning passive smoking	Observational, multilevel, and cross-sectional	Independent variable, original data, only total score	Light smokers are less likely to have houses and cars in which smoking is allowed, to have visited drinking or eating establishments that allow smoking, and to be systematically exposed to tobacco smoke in the workplace	No limitations reported about the TCS as a variable	Advocacy
Kovess et al.^16^ 2013, France	To ascertain patterns of parental smoking in the vicinity of children in Eastern and Western Europe and their relation to TCS scores	Observational, multilevel, and cross-sectional	Independent variable, original data, only total score	Eastern European parents were about twice as likely to smoke near their children as those in Western Europe. Current maternal smoking prevalence was similar. A strong relationship was observed between parental education, tobacco control policies, and smoking near the child	No limitations reported about the TCS as a variable	Advocacy
Willemsen et al.^20^ 2012, the Netherlands	To examine how a country’s level of tobacco control is associated with markers of denormalization of smoking, smoking prevalence, and societal support for tobacco control	Observational, ecological, and cross-sectional	Dependent and independent variable, original data, only total score	Smokers in EU countries with higher TCS scores are more concerned about the effect of smoking. Support for tobacco policies is higher in countries with more concerned smokers	No limitations reported about the TCS as a variable	Advocacy and research
Bogdanovica et al.^25^ 2011, UK	To test the hypothesis that smoking prevalence is higher in countries with high levels of public sector corruption and explore the ecological association between smoking prevalence and a range of other national characteristics in current EU Member States	Observational, ecological, and cross-sectional	Dependent variable, original data, total score and by components	Smoking prevalence was significantly higher in countries with higher scores for corruption, material deprivation, and gender inequality, and lower in countries with higher gross domestic product per capita, social spending, life satisfaction, and human development scores	No limitations reported about the TCS as a variable	Advocacy
Martínez- Sánchez et al.^21^ 2010, Spain	To describe the correlation between the TCS and smoking prevalence, self-reported exposure to SHS, and attitudes towards smoking restrictions in the 27 countries of the EU	Observational, ecological, and cross-sectional	Independent variable, original data, total score and by components	A direct non-significant association was found between TCS scores and the prevalence of smoke-free houses, and a non-significant inverse correlation with allowing smoking in certain rooms	No limitations reported about the TCS as a variable	Advocacy
Tual et al.^17^ 2010, France	To explore the relationship between SHS exposure and the strength of national-level tobacco control policies	Observational, multilevel, and cross-sectional	Dependent variable, original, data only total score	The Carbone monoxide concentration decreased with the strength of tobacco control policies, as scored by the TCS in a large non-smoker European population	No limitations reported about the TCS as a variable	Advocacy and research
Hublet et al.^9^ 2009, Belgium	To investigate smoking policies in 29 European countries in relation to the national smoking prevalence among young people	Observational, multilevel, and cross-sectional	Independent variable, original data, total score and by components	3.8% variance in regular smoking in boys and 3.5% in girls can be attributed to country structure or country of residence. In boys, this variance is associated with country-level tobacco control	No limitations reported about the TCS as a variable	Advocacy
Schaap et al.^11^ 2008, the Netherlands	To examine the extent to which tobacco control policies correlate with smoking cessation	Observational, ecological, and cross-sectional	Independent variable, original data, total score and by components	High-educated smokers were more likely to quit smoking in all age-sex groups. TCS score was positively associated with quit ratios in all age-sex groups, with no consistent differences between high and low education	The information described by the TCS score is about policies in 2005 and recently implemented policies are not incorporated; therefore, the impact of such policies may be underestimated when using the current version of the TCS	Advocacy

**Table 3:** Main characteristics of the studies that used the TCS from a secondary source and estimated the scores of the countries using TCS methodology (N=10)

*Reference, location*	*Objective*	*Study design*	*Use of TCS (Type of variable, original data, total score and/or components)*	*Main results*	*Limitations*	*Conclusions*
So et al.^26^ 2019, UK	To describe changes in smoking prevalence over time within EU member states from 2009–2017; to describe how within-country and between-country variations in the implementation of tobacco control policies are associated with current smoking in individuals; and to describe how these variations affect individuals of different socioeconomic positions.	Observational, multilevel, and longitudinal	Independent variable, not original data, only total score	A general trend of decreasing smoking prevalence over the last decade was found in the EU. There was significant variation at the country level and country-year level, indicating that countries differed significantly in their smoking prevalence trajectory. Strong tobacco control policies were significantly associated with lower odds of being a current smoker, with a greater effect in upper class occupations	No limitations reported about the TCS as a variable	Research
Serrano- Alarcón et al.^27^ 2019, Portugal	To evaluate the impact of tobacco control policies on smoking among older adults in Europe from 2004–2013	Observational, multilevel, and longitudinal	Independent variable, not original data, total score and by components	A 10-point increase in the TCS score was associated with a drop in the probability of smoking by 1.1 percentage points (not significant). Pricing and smoke-free policies were significantly associated with smoking	No limitations involving the use of TCS	Research
Bosdriesz et al.^31^ 2016, the Netherlands	To assess whether tobacco control policy was associated with socioeconomic inequalities in smoking across the EU in the period 2006– 2012	Observational, multilevel, and longitudinal	Independent variable, not original data, total score and by components	An association was found between tobacco control policies and smoking cessation among higher educated smokers. In middle- and high-educated smokers, policies were also associated with a decrease in smoking intensity	No limitations involving the use of TCS	Advocacy
Bosdriesz et al.^32^ 2015, the Netherlands	To assess whether developments in tobacco control policy in the Netherlands were associated with smoking cessation and smoking intensity.	Observational, multilevel, and longitudinal	Independent variable, not original data, only by components score	Progress in tobacco control policy in the Netherlands was significantly associated with an increase in the quit ratios (2001–2011) but were not significantly associated with smoking intensity among smokers. The strength of the associations was similar for low- and high-education groups	No limitations reported about the TCS as a variable	Advocacy and research
Bosdriesz et al.^30^ 2015, the Netherlands	To assess variations in the progress of tobacco control policy development in Europe and to identify whether the variations can be decomposed into specific patterns or components	Observational, ecological, and longitudinal	Dependent variable, not original, only by components score	Progress in tobacco control policy development in Europe was not uniform. Consistent progress was observed in several areas but was lacking in tobacco prices and smoking cessation support	a. TCS score sometimes fails to express the degree to which policies are enforced; b. some policy areas could not be quantified readily; c. not able to include each separate measure of the TCS in its own right	Research
Klumbiene et al.^29^ 2015, Lithuania	To evaluate the association between tobacco control policies and trends in smoking cessation in Lithuania in 1994–2010	Observational, ecological, and longitudinal	Independent variable, not original data, total score and by components	Great progress in the development of tobacco control policy has been achieved in Lithuania. This progress was associated with an increase in smoking cessation. This association was stronger among younger than older people	No limitations reported about the TCS as a variable	Research
Kuipers et al.^28^ 2015, the Netherlands	To examine the association between tobacco control policies and adolescent smoking, and to investigate the differences in this association between adolescents of high and low socio-economic status (SES)	Observational, multilevel, and cross-sectional	Independent variable, not original data, only total score	Adolescent smoking prevalence rates were higher among low-SES respondents than their high-SES peers. Stronger national-level tobacco control policies were associated with lower odds of daily smoking	The TCS used in the current study contains five domains of tobacco control, not all of which may be as likely to affect adolescent smoking	Advocacy and research
Bosdriesz et al.^6^ 2015, the Netherlands	To provide insight into the role of political factors in the development of tobacco control policy over time	Observational, ecological, and longitudinal	Dependent variable, not original, total score and by components	An association was found between left-wing government and TCS over the period 1996–2003, but not over the whole studied period (1996–2010). The association between government effectiveness and TCS was significant and negative over the whole period, but positive between 2001 and 2005	The TCS contains little information on their enforcement in practice	Advocacy
Movsisyan et al.^33^ 2014, Armenia	To measure the 5-year progress in the implementation of FCTC in Armenia	Observational, ecological, and cross-sectional	Dependent variable, not original, total score and by components	The estimated TCS score for Armenia for smoke-free public places, advertising ban, health warnings, and treatment are below the European average (2005–2007). However, the score estimate for price and public spending are above average	a. Potential measurement error; b. inadequate accuracy and comparability of data; c. the estimates could have been affected by exchange rate fluctuations	Advocacy and research
Heydari et al.^34^ 2012, Iran	To obtain an overview of tobacco control strategies in the Eastern Mediterranean region	Observational, ecological, and cross-sectional	Dependent variable, not original, total score and by components	Afghanistan scored highest for tobacco pricing. Oman scored higher than others for regulations and enforcement of bans on smoking in public places. The Islamic Republic of Iran had the top score on budgeting for tobacco control activities, in prohibition and enforcement of tobacco advertising, and placement of health warnings on cigarette packets. Syrian Arab Republic, Tunisia, and Kuwait had the best provision of smoking cessation services, whereas Pakistan, Saudi Arabia, Somalia, and Yemen scored zero	As the data were extracted from sources such as MPOWER measures and the Tobacco Atlas, they may not cover all important variables and the results may not be conclusive	Research

Twelve out of 21 articles (60%) using individual TCS scores included all six policy components in the analysis. The most frequently used policy components were the individual score on bans in public places (n=20; 95.2%) and advertising bans (n=16; 76.2%). In contrast, the least used were data on public spending on information campaigns (n=12; 57.1%). The publications included data from between 1 and 31 countries; only one publication used scores from all of the countries included in the TCS report, including >30 EU and non-EU countries^7^; however, 46.9% of publications included scores from all EU Member states except Croatia because it was first included in 2013.

Half (n=16) of the publications were directed towards policymakers with the aim of urging governments to implement more stringent tobacco control policies, 6 publications aimed to foster further research on this topic (18.8%), and the conclusions of 10 papers (31.3%) addressed both aims.

### Articles using TCS scores from primary reports

Almost all of the studies that used the TCS reports as a primary source (n=22) (Table 2), were observational in nature (n=21; 95.5%) and 19 were cross-sectional (86.4%). According to the type of unit of analysis, half were ecological studies and half multilevel. These studies aimed to address the relationship between tobacco control policies and several outcomes, such as the prevalence of preterm births and low birthweight^7^, of hard-core and light smokers^8^, and of smoking in adolescents^9,10^; smoking prevalence and quit ratios^4,11^; consumption of rolling tobacco, e-cigarettes, and readiness to quit in adults^12^; and risk of lung cancer^13^. Other indicators were smoking in private venues^1,14^, self-reported exposure to secondhand smoke (SHS)^15-17^, and attitudes towards smoking and tobacco product restrictions^18-21^.

Other publications were focused on exploring the association between the price of tobacco products and infant mortality^22^, the effects of sales restriction laws on adolescents^23^, and the factors associated with exposure to tobacco and e-cigarette advertising^24^. One study assessed the association between smoking prevalence and public sector corruption and other national characteristics^25^. The main characteristics and results of each article are given in Table 2.

### Articles using TCS methodology to compute new scores

As shown in Table 3, ten studies calculated new scores to measure tobacco control policies at a country level in a particular year using the TCS rationale and methodology instead of the original TCS. Most of these studies used data from European countries with a longitudinal design aimed at assessing the association between tobacco control policies and smoking^6,26,27^ and socio-economic inequalities outcomes in adolescents^28^ or adults over time^29-31^, or to examine political factors that drive tobacco control policy development^32^. According to the type of data, these publications were half ecological and half multilevel studies.

Two publications computed scores for nonEuropean countries to monitor their tobacco control policy implementation by using the same rationale and methodology. These publications were aimed at measuring the progress after implementation of the WHO-FCTC in Armenia^33^ and providing an overview and comparing the tobacco control progress in Eastern Mediterranean countries^34^. The main characteristics and results of each study are shown in Table 3.

### Main limitations of the TCS mentioned by the studies

Only 11 (34.4%) of all publications included comments on the limitations of using the TCS score as a tool to assess tobacco control policies. The main limitations reported by the studies were that they failed to express the degree to which legislative policies are enforced^6,32,35^, except for the smoke-free policies^4^. Another limitation is that the countries’ rankings have only slightly changed over the years (i.e. the UK has remained in the top position from 2007 to 2016)^4^. This low variance across countries may reduce the robustness of the results of the studies^10^. Moreover, some studies reported that the information described by the TCS score(s) does not incorporate the most recent national legislation on tobacco control due to its cross-sectional design^13^, potentially underestimating the impact of such policies when using the TCS^11^.

Finally, among the studies not using data from the original TCS reports, the main limitations were that some policy areas could not be quantified accurately and that some of the policy components assessed by the TCS could not be included^34^ because of potential error in the measurement of their estimates, and inadequate accuracy and comparability of the data^33^.

## DISCUSSION

Our results reveal that the TCS has been used mostly in observational, cross-sectional studies with either ecological (country as the unit of analysis) or multilevel data (individual data from surveys as the first-level unit and TCS score by country as secondlevel aggregated information). The TCS score has been mainly used as an independent variable to explain the potential variation in outcomes (i.e. tobacco product use, exposure to SHS, attitudes towards legislation, etc.), and mostly employed in European countries, as these countries were the target of the TCS when it was created.

### Interpretation of the results

This is the first attempt to assess all of the available publications that have used the TCS as a means to measure tobacco control policy implementation since it was developed in 2006. In addition, this is the first study to map out the characteristics of the use of the TCS in scientific research, to understand how this tool has been applied despite its original design as a means to advocate for comprehensive tobacco control policies. Therefore, our findings suggest that the TCS has commonly been used as an indicator of the state of tobacco control policies in Europe.

Almost all of the studies assessed tobacco control policies through the total TCS score, and most have used the policy components scores from the primary published reports. The policy components most commonly studied were public smoking bans and tobacco product advertising bans, possibly because they are two of the measures that have been most frequently regulated in Europe since the WHO-FCTC came into force in 2005^36^.

Another important issue to address is the crosssectional and temporal comparability of the TCS because most of the studies make comparisons across countries and/or over time. Notably, Joossens and Raw^5^ designed the scale to compare tobacco control policies across countries at a particular time point. Thus, the reference values for scores are sustained across each report. However, these scores are not comparable across years because these standards have changed over time (i.e. the weighted average price for cigarettes was €8.5 in 2013 and €10 in 2016, considering the EU average Purchasing Power Standard)^37,38^; on the other hand, the scale methodology and scoring system changed between 2007 and 2010^39^. Consequently, longitudinal studies to ensure temporal comparability between and within countries require adjusting scores to the highest standards by an escalation process and re-calculating scores from the 2005 and 2007 reports using the newest scoring system and methodology.

Importantly, most of the studies with a longitudinal design conducted in Europe have adapted the scale rationale and methods to estimate the level of implementation of tobacco control policies to ensure the temporal comparability and include data about years for which the TCS had not been published^26,27,29,31,32^. Few non-European countries have adapted the scale as a proxy to monitor the status of tobacco control policies in non-European countries^33,34^. Unfortunately, most of these studies did not clearly explain how they adapted the TCS to estimate new score(s) for each policy component. Therefore, new studies should provide a full description of their adaptation process and the potential limitations and strengths not only to ensure its replication, but also to further develop strategies to adapt the TCS to other contexts overseas.

These results highlight that the TCS, regardless of its limitations, has been applied as an objective indicator to measure the strength of the implementation of tobacco control policies at the country level. Other studies have used a total score obtained from summing the scores (from 1 to 5) assigned to each MPOWER policy dimension in the WHO’s Reports on the Global Tobacco Epidemic^40,41^. MPOWER’s composite score has some clear advantages over the TCS total score because it is available for all countries, not only European countries, and is comparable over time. However, this proxy also has some disadvantages for research purposes. First, it assigns the same weight to each of the six individual MPOWER scores without taking into consideration that some MPOWER measures have been proven to be more effective than other measures (i.e. taxation). Second, MPOWER’s composite score has a narrower score range than the TCS score (6–29 vs 0–100, respectively), which limits variation across countries and may make it difficult to address variability between countries. Finally, unlike the MPOWER composite score, the TCS score is not affected by the government’s political agenda, as the TCS is built on information from objective databases (i.e. Eurostat) and the Civil Society.

More than 65% of the reviewed publications did not report any limitation of the TCS as a proxy for measuring tobacco control policies. Nonetheless, Joossens and Raw^5^ already reported some of its major limitations, including difficulties in assessing enforcement versus implementation and its critical dependence on tobacco control experts’ judgement when scoring^5^. Therefore, such underreporting of limitations makes it difficult to fully describe the limitations that researchers encounter, which is indispensable to moving forward in the field. Among the articles reporting limitations, most of them highlighted the fact that the TCS score(s) did not measure the enforcement of policies except smokefree policies. In this sense, no previous studies have examined the disparity between the implementation and enforcement of tobacco control policies; however, the TCS being predictive of so many outcomes suggests that the implementation of these policies is a good proxy of enforcement. In addition, some studies have questioned the ability of the TCS to incorporate new policies, such as smoke-free outdoor policies, indicating that the authors of the TCS should discuss how to incorporate these new tobacco control policies and which weight they should have in the scale.

Moreover, our study shows that the TCS has been commonly used in Europe over the last decade, but three research groups from Spain, the Netherlands, and the UK account for more than half of the publications using the TCS for research purposes. This suggests that these groups have led and consolidated the use of this monitoring tool in the tobacco control research field. This is supported by the fact that the publications conducted by these three research groups have received a higher number of citations, including a paper^13^ with 54 citations and another paper^23^ with 103 citations (in Web of Science) up to December 2019.

More than half of all publications directed their conclusions towards advocacy for improving tobacco control policies. Therefore, most authors find the TCS useful for linking data to policy action, even though the TCS has been commonly used for research purposes. Therefore, the TCS has not lost its intended original purpose for advocacy, as it was developed to detect areas of improvement within each country and to establish comparisons among countries through a ranking, in order to motivate governments to strengthen their weakest polices^5^.

Our results indicate that, despite its potential limitations and lack of a formal validity assessment, the TCS is a good proxy of the strength of tobacco control policies implementation, or at least the best approximation developed so far. However, the TCS has been used at face-value. No attempts have been made to formally validate the scale. Construct validity of the TCS is a complex issue given the composite structure of the TCS itself, though some dimensions are based on objective data (i.e. price and SHS exposure) from population-based surveys and reports of the European Commission; others are based on the answers of one or two informants to an ad hoc questionnaire (i.e. cessation budget at national level)^5^.

### Limitations and strengths

Publication bias is a potential source of error when the units of the investigation are published papers^42^. We searched the available literature in PubMed, the main biomedical database, as well as Web of Science and Google Scholar, and checked all references to identify other articles not published in academic journals. However, the possibility that unpublished manuscripts or other documents addressing the topic of interest may have been missed cannot be ruled out, but it was an *a priori* decision made by the experienced research team that was composed of tobacco control and policy experts, including the author of the TCS. Under these circumstances, selection (publication) bias seems unlikely to have affected the study.

Other potential limitations of our study are linked to the fact that a high number of the publications analyzed here did not include any comment about the limitations and strengths of using TCS scores as a variable to monitor tobacco control policy implementation in research. This missed reporting has hindered the identification of the main limitations and strengths of this tool for different types of study designs, outcomes, or statistical analysis; therefore, our study may have some missing information.

However, our study is the first to assess all published articles using the TCS as an indicator of tobacco control policy implementation and to characterize its use in tobacco control research, giving a full and comprehensive overview of how and for which purposes the TCS has been employed in previous studies. This study also presents information on how to best use the TCS as described by the authors in the Limitations sections of the publications. Thus, our study presents the lessons learned from previous research, creating an opportunity for researchers to plan to use the TCS to improve the quality of future studies.

## CONCLUSIONS

This study shows that the TCS has been commonly used in observational, mostly ecological, studies to assess variations in a concrete outcome according to the policies instituted in Europe as a proxy of tobacco control implementation. In addition, the TCS has been employed to detect changes in individual and population outcomes (i.e. smoking prevalence or cessation) and establish conclusions about how policies have an effect in specific populations.

Our recommendations to researchers and policymakers planning to use the TCS in their future research are as follows. First, the TCS scoring methodology needs to be fully understood, as comparability is not ensured among countries across years. Second, researchers should consider a certain time gap between measuring the TCS score and the outcomes, as the TCS may not include the most recently adopted policies and policies need time to have an effect. Third, knowing the limitations of the TCS in measuring implementation (vs enforcement) of tobacco control policies is important. Fourth, researchers need to take into account the low variance of some tobacco control policies across countries, which may also reduce the robustness of the estimates.

A logical next step for future applications of the TCS in research would be to study the impact of tobacco control policy enforcement in terms of several indicators, such as prevalence, SHS, and tobacco sales, and to assess the impact of these policies at the population level. To achieve this goal, more extensive cross-country population-based surveys are needed to include new enforcement measures in future editions of the TCS (i.e. about compliance with smoke-free bans in public places differently than workplaces and hospitality venues, or about advertising, promotion, and sponsorship bans).

Finally, to gain a broader perspective of tobacco control as a public health need and build a stronger tool for tobacco research, we suggest adapting and extending the TCS to other countries of the WHO European Region, and to the reality of other regions of the globe, such as Latin America or Asia, incorporating local and cultural characteristics of these regions while preserving the comparability among countries worldwide.

## CONFLICTS OF INTEREST

The authors have completed and submitted the ICMJE Form for Disclosure of Potential Conflicts of Interest and none was reported.

## FUNDING

AF, MF, CM, and EF were supported by the Ministry of Research and Universities from the Government of Catalonia [2017SGR319]. EF was also supported by the Instituto de Salud Carlos III, Government of Spain, co-funded by the European Regional Development Fund (FEDER) [INT16/00211 and INT17/00103]. CM was also supported by the Instituto de Salud Carlos III, Government of Spain, co-funded by the European Regional Development Fund (FEDER) [INT17/00116] and Ministry of Health from the Government of Catalonia [PERIS No. 9015586920/2017]. We thank CERCA Programme/Generalitat de Catalunya for institutional support.

## AUTHORS’ CONTRIBUTIONS

Study design: AF, AB, CM, and EF. Collected data and prepared database for analysis: AF and AB. Contributed to strategy of analysis: AF, AB, CM, and EF. Analyzed data: AF. Interpreted data results: AF, AB, CM, LJ, MF, AP, and EF. Drafted manuscript: AF. Critically revised manuscript: All authors. Approved final manuscript version: All authors. Guarantors: EF and CM.

## PROVENANCE AND PEER REVIEW

Not commissioned; externally peer reviewed.

## References

[1] Ferketich AK, Lugo A, La Vecchia C (2014). Relation between national-level tobacco control policies and individual-level voluntary home smoking bans in Europe. Tob Control.

[2] Bertollini R, Ribeiro S, Mauer-Stender K, Galea G (2016). Tobacco control in Europe: a policy review. Eur Respir Rev.

[3] International Agency for Research on Cancer (2009). Evaluating the effectiveness of smoke-free policies. IARC Handbook of Cancer Prevention: Tobacco Control.

[4] Feliu A, Filippidis FT, Joossens L (2019). Impact of tobacco control policies on smoking prevalence and quit ratios in 27 European Union countries from 2006 to 2014. Tob Control.

[5] Joossens L, Raw M (2006). The Tobacco Control Scale: a new scale to measure country activity. Tob Control.

[6] Bosdriesz JR, Willemsen MC, Stronks K, Kunst A (2015). Patterns of tobacco control policy progress in 21 European countries. Tob Regul Sci.

[7] Díez-Izquierdo A, Balaguer A, Lidón-Moyano C (2018). Correlation between tobacco control policies and preterm births and low birth weight in Europe. Environ Res.

[8] Feliu A, Fernandez E, Martinez C, Filippidis F (2019). Are smokers ‘hardening’ or rather ‘softening’? An ecological and multilevel analysis across 28 European Union countries. Eur Respir J.

[9] Hublet A, Schmid H, Clays E (2009). Association between tobacco control policies and smoking behaviour among adolescents in 29 European countries. Addiction.

[10] Pförtner TK, Hublet A, Warrer C (2016). Socioeconomic inequalities in the impact of tobacco control policies on adolescent smoking. A multilevel study in 29 European countries. Addict Behav.

[11] Schaap MM, Kunst AE, Leinsalu M (2008). Effect of nationwide tobacco control policies on smoking cessation in high and low educated groups in 18 European countries. Tob Control.

[12] Lidón-Moyano C, Martín-Sánchez JC, Saliba P, Graffelman J, Martínez-Sánchez J (2017). Correlation between tobacco control policies, consumption of rolled tobacco and e-cigarettes, and intention to quit conventional tobacco, in Europe. Tob Control.

[13] González-Marrón A, Martín-Sánchez JC, Miró Q (2019). Relation between tobacco control policies and population at high risk of lung cancer in the European Union. Environ Res.

[14] Martínez-Sánchez JM, Blanch C, Fu M, Gallus S, La Vecchia C, Fernández E (2014). Do smoke-free policies in work and public places increase smoking in private venues?. Tob Control.

[15] Filippidis FT, Agaku IT, Girvalaki C (2016). Relationship of secondhand smoke exposure with sociodemographic factors and smoke-free legislation in the European Union. Eur J Public Health.

[16] Kovess V, Pilowsky DJ, Boyd A (2013). Parental smoking in the vicinity of children and tobacco control policies in the European Region. PLoS One.

[17] Tual S, Piau JP, Jarvis MJ, Dautzenberg B, Annesi-Maesano I (2010). Impact of tobacco control policies on exhaled carbon monoxide in non-smokers. J Epidemiol Community Heal.

[18] Lidón-Moyano C, Sampedro-Vida M, Matilla-Santander N (2018). Attitudes towards tobacco product regulations and their relationship with the tobacco control policies. Prev Med.

[19] Rughinis C, Rughiniş R (2014). Influence of daily smoking frequency on passive smoking behaviors and beliefs. Revista de Cercetare si Interventie Sociala.

[20] Willemsen MC, Kiselinova M, Nagelhout GE, Joossens L, Knibbe R (2012). Concern about passive smoking and tobacco control policies in European countries: An ecological study. BMC Public Health.

[21] Martínez-Sánchez JM, Fernández E, Fu M (2010). Smoking behaviour, involuntary smoking, attitudes towards smoke-free legislations, and tobacco control activities in the European Union. PLoS One.

[22] Filippidis FT, Laverty AA, Hone T, Been J, Millett C (2017). Association of cigarette price differentials with infant mortality in 23 European Union countries. JAMA Pediatr.

[23] Kuipers MAG, Brandhof SD, Monshouwer K, Stronks K, Kunst A (2017). Impact of laws restricting the sale of tobacco to minors on adolescent smoking and perceived obtainability of cigarettes: an intervention-control pre-post study of 19 European Union countries. Addiction.

[24] Filippidis FT, Laverty AA, Fernandez E, Mons U, Tigova O, Vardavas C (2017). Correlates of self-reported exposure to advertising of tobacco products and electronic cigarettes across 28 European Union member states. Tob Control.

[25] Bogdanovica I, McNeill A, Murray R, Britton J (2011). What Factors influence smoking prevalence and smoke free policy enactment across the European union Member States. PLoS One.

[26] So VHT, Best C, Currie D, Haw S (2019). Association between tobacco control policies and current smoking across different occupational groups in the EU between 2009 and 2017. J Epidemiol Community Health.

[27] Serrano-Alarcón M, Kunst AE, Bosdriesz JR, Perelman J (2019). Tobacco control policies and smoking among older adults: a longitudinal analysis of 10 European countries. Addiction.

[28] Kuipers MAG, Monshouwer K, Van Laar M, Kunst A (2015). Tobacco Control and Socioeconomic Inequalities in Adolescent Smoking in Europe. Am J Prev Med.

[29] Klumbiene J, Sakyte E, Petkeviciene J, Prattala R, Kunst A (2015). The effect of tobacco control policy on smoking cessation in relation to gender, age and education in Lithuania, 1994–2010. BMC Public Health.

[30] Bosdriesz JR, Nagelhout GE, Stronks K, Willemsen M, Kunst A (2015). The Association Between Tobacco Control Policy and Educational Inequalities in Smoking Cessation in the Netherlands from 1988 Through 2011. Nicotine Tob Res.

[31] Bosdriesz JR, Willemsen MC, Stronks K, Kunst A (2016). Tobacco control policy and socio-economic inequalities in smoking in 27 European countries. Drug Alcohol Depend.

[32] Bosdriesz JR, Willemsen MC, Stronks K, Kunst A (2015). Tobacco control policy development in the European Union: do political factors matter?. Eur J Public Health.

[33] Movsisyan NK, Connolly GN (2014). Measuring Armenia’s progress on the Tobacco Control Scale: an evaluation of tobacco control in an economy in transition, 2005–2009. BMJ Open.

[34] Heydari G, Talischi F, Masjedi MR, Alguomani H, Joossens L, Ghafari M (2012). Comparison of tobacco control policies in the Eastern Mediterranean countries based on tobacco control scale scores. East Mediterr Heal J.

[35] Allen K, Kypridemos C, Hyseni L (2016). The effects of maximising the UK’s tobacco control score on inequalities in smoking prevalence and premature coronary heart disease mortality : a modelling study. BMC Public Health.

[36] World Health Organization (2017). WHO Report on the Global Tobacco Epidemic, 2017: Monitoring tobacco use and prevention policies.

[37] Joossens L, Raw M (2014). The Tobacco Control Scale 2013 in Europe.

[38] Joossens L, Raw M (2017). The Tobacco Control Scale 2016 in Europe.

[39] Joossens L, Raw M The Tobacco Control Scale 2010 in Europe.

[40] Ngo A, Cheng KW, Chaloupka FJ, Shang C (2017). The effect of MPOWER scores on cigarette smoking prevalence and consumption. Prev Med.

[41] Dubray J, Schwartz R, Chaiton M, O'Connor S, Cohen J (2015). The effect of MPOWER on smoking prevalence. Tob Control.

[42] Moher D, Liberati A, Tetzlaff J, Altman D (2009). The PRISMA Group. Preferred reporting items for systematic reviews and meta-analyses: The PRISMA statement. PLoS Med.

